# Clinical and psychological correlates of health-related quality of life in obese patients

**DOI:** 10.1186/1477-7525-8-90

**Published:** 2010-08-23

**Authors:** Edoardo Mannucci, Maria L Petroni, Nicola Villanova, Carlo M Rotella, Giovanni Apolone, Giulio Marchesini

**Affiliations:** 1 Geriatric Unit, Department of Critical Care, University of Florence, Italy; 2 Department of Metabolic Rehabilitation, San Giuseppe Hospital, Piancavallo, Italy; 3 Unit of Metabolic Diseases & Clinical Dietetics, Department of Clinical Medicine, "Alma Mater Studiorum" University, Bologna, Italy; 4 Endocrine Unit, Department of Clinical Pathophysiology, University of Florence, Italy; 5 Clinical Research Laboratory, "Mario Negri" Institute for Pharmacologic Research, Milan, Italy

## Abstract

**Background:**

Health-related quality of life (HRQL) is poor in obese subjects and is a relevant outcome in intervention studies. We aimed to determine factors associated with poor HRQL in obese patients seeking weight loss in medical units, outside specific research projects.

**Methods:**

HRQL, together with a number of demographic and clinical parameters, was studied with generic (SF-36, PGWB) and disease-specific (ORWELL-97) questionnaires in an unselected sample of 1,886 (1,494 women; 392 men) obese (BMI > 30 kg/m^2^) patients aged 20-65 years attending 25 medical units scattered throughout Italy. The clinics provide weight loss treatment using different programs. General psychopathology (SCL-90 questionnaire), the presence of binge eating (Binge Eating scale), previous weight cycling and somatic comorbidity (Charlson's index) were also determined. Scores on SF-36 and PGWB were compared with Italian population norms, and their association with putative determinants of HRQL after adjustment for confounders was assessed through logistic regression analysis.

**Results:**

HRQL scores were significantly lower in women than in men. A greater impairment of quality of life was observed in relation to increasing BMI class, concurrent psychopathology, associated somatic diseases, binge eating, and weight cycling. In multivariate analysis, psychopathology (presence of previously-diagnosed mental disorders and/or elevated scores on SCL-90) was associated with lower HRQL scores on both psychosocial and somatic domains; somatic diseases and higher BMI, after adjustment for confounders, were associated with impairment of physical domains, while binge eating and weight cycling appeared to affect psychosocial domains only.

**Conclusions:**

Psychopathological disturbances are the most relevant factors associated with poor HRQL in obese patients, affecting not only psychosocial, but also physical domains, largely independent of the severity of obesity. Psychological/psychiatric interventions are essential for a comprehensive treatment of obesity, and to improve treatment outcome and to reduce the burden of disease.

## Introduction

Obesity is associated with impairment of health-related quality of life (HRQL) in psychological, social, and physical domains [1,2]. Improvement of HRQL is recognised as a relevant measure of treatment outcome in obese patients, both in medically- [3,4] and surgically-treated cases [1,2]. The specific HRQL concepts that relate to obesity are not clearly defined, although several aspects of patients' lives are relevant to obesity [3,4]. Factors reported to be associated with greater impairment of quality of life among treatment seeking obese patients include female sex [5,6], higher body mass index [7,8], binge eating disorder [9,10] and psychopathology [9]. They are often associated in the same individuals. For this reason, the assessment of the relative contribution of each condition to HRQL can only be attempted with a large sample size. In particular, the relative role of somatic diseases, psychological distress and previous unsuccessful dieting has never been clearly defined. A few studies found that psychological distress is also affecting physical domains to a greater extent than somatic disorders [9]. A correct identification of factors associated with poor HRQL is essential to develop strategies to improve outcome in these patients, and the association of poor HRQL with depressive symptoms is the rationale for intensive psychological support [11].

The QUOVADIS Study [12] is a multicenter, collaborative survey designed to assess determinants of quality of life in treatment-seeking obese patients. The survey collected a lot of patient-reported data, including those more frequently associated with poor HRQL [3], in a large sample of obese subjects seeking weight-reducing programs in 25 medical Italian hospital-based clinics for the treatment of obesity. Thus, the QUOVADIS database provides a unique opportunity to investigate the factors associated with poor HRQL, to be used as a guide for treatment outcome [13].

We aimed to identify the factors associated with poor HRQL in obese subjects, with special reference to the possible role of psychological distress and psychiatric comorbidity which might make psychological support essential to improve treatment outcome.

## Sample and methods

### Participating subjects with obesity

The philosophy of the QUOVADIS study and the general characteristics of the population have been partly published in a previous report [12]. Briefly, the study enrolled a representative sample of patients attending 25 hospital-based clinics for weight loss throughout the country. The centers were both outpatient and inpatient specialized obesity clinics, providing multidisciplinary programs for weight loss. The subjects were consecutively enrolled to exclude selection bias. At enrolment, they were interviewed as to weight history, previous somatic and mental diseases, hospital admission during the previous year, self-evaluation of physical activity and eating pattern, and completed a set of self-administered questionnaires. In addition, they were submitted to routine blood tests, but these data were not used in the present report, specifically based on self-awareness of previous disorders. We report an analysis based on 1886 subjects whose complete data on the Case Report Form and on questionnaires were available.

The weight history was checked according to a pre-defined structured interview [14]. Patients' answers were used to compute the total number of dieting programs, and the total weight loss induced by dieting programs. The number of dieting attempts was normalized for the time since first dieting; all other parameters of diet history were normalized for time since age 20.

To facilitate handling of data, the Case Report Forms were implemented in an extranet database provided by CINECA (Casalecchio di Reno, Italy), an Interuniversity Consortium of 15 Italian Universities, using the AMR (Advanced Multicenter Research) methodology, which allows the management of the whole research using standard web-browsers.

All subjects signed an informed consent to take part in the study, which was approved by the ethical committees of the individual centers, after approval by the committee of the coordinating center (University of Bologna)

### Measures

Quality of life was measured using 3 different tools. The Obesity-Related Well-Being questionnaire (ORWELL-97), an obesity-specific tool, was used with the specific aim to collect data useful in a longitudinal evaluation of HRQL following treatment [15]. It measures the intensity and the subjective relevance of physical and psychological distress generated by overweight.

A score in the ORWELL-97 questionnaire ≥ 70, corresponding to the 75° percentile of the population, was considered indicative of a clinically significant burden of obesity on HRQL.

The Medical Outcome Survey Short-Form 36 (SF-36) was used as a generic measure of HRQL, with the specific aim to measure the extent of the defect in HRQL in both physical and mental domains [16]. The questionnaire is specifically constructed to measure the full range of health status and well-being by means of 36 multiple-choice questions. It measures 8 different domains, 4 in the area of physical health (Physical Functioning, Role Limitation-Physical, Bodily Pain, General Health) and 4 in the area of mental health (Role Limitation-Emotional, Vitality, Mental Health, and Social Functioning). It has been extensively validated worldwide and Italian normative values have been defined [17].

The Psychological General Well-Being (PGWB) questionnaire was used to score psychological distress [18]. The responses to 22 questions are arranged in 6 affective states: anxiety, depressed mood, positive well-being self-control, general health and vitality. The Italian version of the questionnaire has been recently validated and normative values are available to compare the results with population standards [19].

For both SF-36 and PGWB, the values of individual domains of each patient were compared to the age- and sex-matched Italian population norms [17,19] using the Z-score (difference between patient value and control mean, divided by control standard deviation). According to Cohen [20], the average Z-scores (effect sizes) were rated as small (between 0.20 and 0.50), as moderate (between 0.50 and 0.80) or as large (> 0.80). This proposal is supported by clinical studies [21].

The Binge Eating Scale was used to detect binging [22]; values in the range 17-26 were considered suspect of binge eating, whereas values ≥ 27 were taken as predictive of Binge Eating Disorder. This classification was used to score binge eating on a scale from 0 (< 17) to 2 (≥ 27).

The Symptom Check List-90 questionnaire was used to identify subjects with a psychopathological profile [23]. A value ≥ 1 in the Global Severity Index (GSI) is suggestive of psychopathology, scored as mild (1.00 - 1.49), moderate (1.50 - 1.99), or severe (≥ 2.00). These results of SCL-90 were combined with clinical data to score the presence of mental disorder on a scale from 0 to 5. A previous diagnosis of psychopathological problems was valued 2 points, GSI values in the range 1.00-1.49 (mild distress) were given a score of 1, values between 1.50 and 1.99 (moderate distress) were given a score of 2, values ≥ 2.00 (severe distress) were given a score of 3.

The presence of somatic diseases was used to calculate a composite score, according to Charlson et al [24], with modifications. For this purpose, one point was added for the reported presence of any of the following states: diabetes, hypertension, other endocrine disorders, liver or biliary disease, hip or knee pain. The presence of cardiovascular disease (any condition, including angina, previous myocardial infarction or stroke, peripheral or carotid vascular disease) and a previous diagnosis of cancer were given 2 points.

Weight history was defined at interview on the basis of body weight at the age of 20 years, age at first dieting and the number of times patients had lost weight as an effect of dietary programs, and scored according to previously-published cut-offs [14]. One point was assigned for any value exceeding the 75° percentile in 3 items reflecting weight history: a) number of dieting attempts (cut-off, 0.56/year); b) weight gain since age 20 years (cut-off, 1.87 kg/year); c) cumulative weight loss (cut-off, 2.63 kg/year).

### Statistical analysis

A first descriptive analysis was carried out on all tested variables. Scores of HRQL (and their relative Z-scores) were grouped according to sex, age, clinical status, complications of disease and eating behavior disorders, and the means and 95% confidence intervals for each patient group and for each domain were calculated.

Differences between obese classes were tested using unpaired t test or Mann-Whitney or Kruskall-Wallis test, due to non-gaussian distribution of data, as appropriate. Differences in the prevalence of categorical data were tested by R × C χ^2 ^test.

Multivariate logistic regression analyses were run using dichotomized Z-scores on individual domains of SF-36 and PGWB as dependent variables. The cut-off value vas set at -1.0, but a sensitivity analysis, using the cut-offs of -0.5 and -1.5 was also performed, and the results were qualitatively confirmed (not reported in details). In the ORWELL-97 model, the dependent variable was an ORWELL score >70. Independent variables were BMI classes, the scores of somatic and mental diseases, the BES grade, and the score of weight history. All models were adjusted for age, gender and BMI.

The Variance Inflation Factor was calculated to assess correlation between independent variables and to exclude multicolinearity.

## Results

### Clinical and psychological characteristics of the study sample

Of the 1,886 patients (1,494 women and 392 men) included in the analysis, 723, 529, and 634 had obesity class I, II and III, respectively. Their age ranged from 20 to 65 years (Class I, 45.4 ± SD 11.3 years; Class II, 44.8 ± 10.7; Class III, 43.9 ± 10.9; P = 0.049, Kruskall-Wallis test). Subjects in Class I were characterized by a higher educational status (primary school 16%, degree 10%) compared with Class II (16% and 9%, respectively) and Class III (21% and 5%, respectively; P < 0.0001). No differences were observed in civil status (single/divorced vs. married/cohabitating or widowed). A larger proportion of subjects in Class III were either housewives (26%) or unemployed (4.4%) compared with Class II (19 and 3.5%) or Class I (17 and 2.8%, respectively; P < 0.0001). Patients in higher classes of obesity showed a significantly greater prevalence of several concurrent illnesses, such as diabetes, hypertension, biliary diseases, and osteoarticular problems, but not of hyperlipidemia, coronary heart and peripheral vascular disease, thyroid disorders, or previously diagnosed psychopathological distress (Table 1).

**Table 1:** Prevalence of physical problems, as reported by patients entering a weight-reducing program. (prevalence and 95% CI)

Clinical data	Class I Obesity BMI, 30-34.9 kg/m^2^ n = 723	Class II Obesity BMI, 35-39.9 kg/m^2^ n = 529	Class III Obesity BMI, ≥40 kg/m^2^ n = 634	P*
Diabetes	5.5 (4.0 - 7.4)	8.2 (6.1 - 10.8)	14.4 (11.8 - 17.3)	< 0.001
Hypertension	25.9 (22.8 - 29.2)	38.6 (34.4 - 42.7)	46.9 (43.0 - 50.7)	< 0.001
Hyperlipidemia	24.0 (21.0 - 27.2)	22.5 (19.0 - 26.1)	21.4 (18.3 - 24.6)	0.506
Coronary heart disease	2.2 (1.3 - 3.5)	3.2 (1.9 - 4.9)	2.5 (1.5 - 4.0)	0.556
Myocardial infarction	1.5 (0.8 - 2.6)	1.3 (0.6 - 2.6)	1.3 (0.6 - 2.4)	0.916
Peripheral vascular dis.	0.0 (0.0 - 0.4)	0.9 (0.3 - 2.0)	0.2 (0.0 - 0.8)	0.530
Gallstones	10.3 (8.3 - 12.7)	13.3 (10.6 - 16.3)	18.0 (15.2 - 21.1)	< 0.001
Cholecystectomy	6.6 (5.0 - 8.6)	8.1 (6.0 - 10.6)	11.6 (9.3 - 14.3)	0.004
Hip pain	27.5 (24.3 - 30.7)	30.7 (26.9 - 34.6)	35.0 (31.3 - 38.7)	0.011
Knee pain	35.9 (32.4 - 39.4)	38.4 (34.3 - 42.5)	47.9 (44.0 - 51.8)	< 0.001
Other endocrine diseases	14.1 (11.7 - 16.7)	17.4 (14.3 - 20.8)	15.5 (12.8 - 18.5)	0.268
Previous cancer	7.2 (5.5 - 9.2)	8.6 (6.5 - 11.2)	5.7 (4.1 - 7.7)	0.152
Psychological distress	17.2 (14.6 - 20.1)	18.3 (15.2 - 21.8)	19.0 (16.1 - 22.2)	0.699

Data are presented as prevalence and 95% CI.

*Chi^2 ^test.

The large majority of subjects reported previous attempts to lose weight (Table 2). Patients with higher BMI reported earlier age of first dieting, greater BMI at age 20 years, higher maximum weight loss obtained in the past, and higher cumulative weight loss per year. Scores on the Binge Eating Scale (BES) were in a range suggestive of binge eating in over one fourth of subjects, while over 10% of patients had BES scores indicative of binge eating disorder. Mean BES scores were significantly higher in patients with class III obesity when compared with the rest of the sample. Similarly, psychopathological distress (Symptom CheckList-90) was more frequent and more severe with progressive obesity class.

**Table 2:** Weight history, scores on the Binge Eating Scale and Symptom CheckList-90 by obesity classes.

	Class I Obesity^†^ n = 723	Class II Obesity n = 529	Class III Obesity n = 634	P value
**Weight history variables**				
BMI at age 20 (kg/m^2^)	23.8 ± 3.4	25.7 ± 4.6	28.3 ± 6.1	< 0.001*
Extra weight since age 20 (kg/year)	1.1 ± 0.8	1.4 ± 0.9	2.2 ± 1.7	< 0.001*
No. of previous dieting (per year)	0.20 (0 - 2.6)	0.21 (0 - 4.0)	0.27 (0 - 2.5)	< 0.001*
Age at first dieting (years)	29.6 ± 11.7	27.1 ± 11.2	25.4 ± 10.4	< 0.001*
Maximum weight loss (kg)	13.0 ± 8.4	15.9 ± 9.1	21.1 ± 11.5	< 0.001*
Cumulative weight loss (kg/year)	1.4 ± 1.9	1.9 ± 2.4	2.7 ± 3.1	< 0.001*
**Binge Eating Scale**				
Score	12.9 ± 9.0	15.0 ± 9.5	16.8 ± 9.5	< 0.001*
Score in the range 17 - 26 (%)	24 (20 - 26)	27 (24 - 31)	29 (25 - 32)	0.064°
Score > 26 (%)	11 (9 - 13)	13 (11 - 16)	15 (13 - 18)	0.042°
**Symptom CheckList-90**				
Global Severity Index	0.70 ± 0.53	0.79 ± 0.57	0.90 ± 0.62	< 0.001*
Mild distress (%)	15 (13 - 18)	14 (12 - 18)	20 (17 - 23)	0.016°
Moderate distress (%)	5 (3 - 6)	9 (7 - 12)	10 (7 - 12)	0.001°
Severe distress (%)	3 (2 - 5)	4 (3 - 6)	6 (5 - 8)	0.024°

Data are presented as mean ± SD, median and range, or as prevalence (95% confidence interval) of cases exceeding selected cut - offs.

^† ^For ranges of obesity classes, see Table 1.

*Kruskall-Wallis or °chi^2 ^test

### Health-related quality of life

HRQL was progressively impaired with increasing BMI. This was shown by all three HRQL measures, i.e., both by the specific ORWELL-97 questionnaire and by the generic SF-36 and PGWB instruments (Table 3). Although all domains were affected, the greatest decrease was observed in domains reflecting physical status, with a less significant impairment in mental health.

**Table 3:** Scores of health-related quality of life in the QUOVADIS population.

	Class I Obesity^†^ n = 723	Class II Obesity n = 529	Class III Obesity n = 634	P*
**ORWELL-97**	41.3 ± 25.7	50.0 ± 28.3	58.5 ± 29.4	< 0.001
**Short Form-36**				
Physical Functioning	76.8 ± 19.5	70.5 ± 21.5	57.1 ± 24.4	< 0.001
Role physical	68.6 ± 35.6	60.3 ± 39.4	51.4 ± 39.9	< 0.001
Bodily pain	64.7 ± 26.7	61.7 ± 27.7	52.8 ± 27.9	< 0.001
General health	61.1 ± 20.9	57.8 ± 20.5	50.3 ± 21.6	< 0.001
Vitality	53.4 ± 19.7	51.2 ± 20.4	47.2 ± 22.0	< 0.001
Role Emotional	65.0 ± 38.5	59.8 ± 39.7	56.4 ± 40.2	< 0.001
Mental health	61.2 ± 21.2	60.8 ± 20.6	58.9 ± 21.1	< 0.001
Social functioning	69.4 ± 24.8	65.5 ± 25.2	61.6 ± 26.9	< 0.001
**Psychological General Well-Being**				
Depressed mood	11.9 ± 2.6	11.5 ± 2.7	11.0 ± 3.2	0.002
Anxiety	15.9 ± 4.9	15.4 ± 5.1	14.9 ± 5.3	< 0.001
Positive well-being	10.6 ± 3.8	10.2 ± 3.9	9.6 ± 4.0	< 0.001
Self-control	11.1 ± 3.1	10.7 ± 3.2	10.3 ± 3.5	< 0.001
General health	10.5 ± 2.7	9.9 ± 2.8	8.9 ± 2.9	< 0.001
Vitality	11.8 ± 3.9	11.3 ± 4.0	10.5 ± 4.0	< 0.001
Global index	71.8 ± 17.3	68.9 ± 18.3	65.3 ± 19.6	< 0.001

Data are reported as means ± SD.

^† ^For ranges of obesity classes, see Table 1.

* Kruskall-Wallis test

The Z-scores on SF-36 domains, reflecting the impairment of HRQL in comparison with sex- and age-specific population norms, showed that HRQL was particularly poor in the domain of Physical Functioning (-1.33), all other domains being in the moderate range (Role-Physical, -0.67; General Health, -0.61; Vitality, -0.61; Social Functioning, -0.57; Bodily Pain, -0.54) or in the small range (Role-Emotional, -0.47; Mental Health, -0.30). The Z-scores on all domains of PGWB, except Vitality (-0.51), were indicative of a small defect (Anxiety, -0.27; Depression, -0.30; Well-Being, -0.35; Self-Control, -0.41; General Health, -0.44).

**Figure 1:**
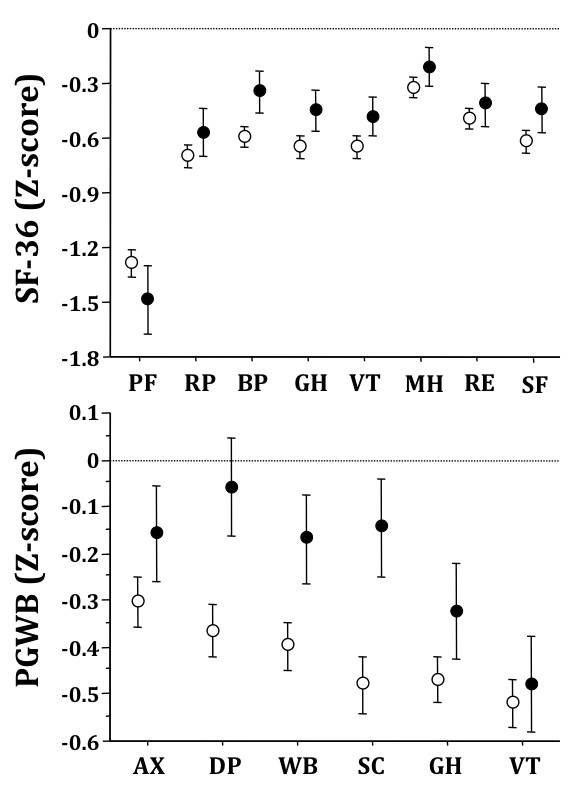
**Z-scores on Short Form-36 (upper panel) and Psychological General Well-being questionnaires in relation to gender (Females, open circles; Males, closed circles)**. Data are presented as means and 95% confidence intervals. All domains crossing the zero line are not significantly different from population norm. Legend for SF-36: PF, Physical Functioning; RP, Role limitation - Physical; BP, Bodily Pain; GH, General Health; VT, Vitality; MH, Mental Health; RE, Role limitation - Emotional; SF, Social Functioning. Legend for PGWB: AX, Anxiety; DP, Depression; WB, Well-Being; SC, Self-Control; GH, General Health; VT, Vitality.

SF-36 and PGWB Z-scores in women and men are summarized in Figure 1. There was a systematic trend towards lower Z-scores in females (by 0.1 - 0.2 points), with the notable exception of Physical Functioning, which was significantly lower in males (-1.49 vs. -1.29 in females; P = 0.025). The difference between males and females was particularly significant in PGWB domains (P < 0.001 for Depression, Self-control, Well-being and General health; < 0.05 for Anxiety; Mann-Whitney U test). Depression was not different from population norm in males.

**Figure 2:**
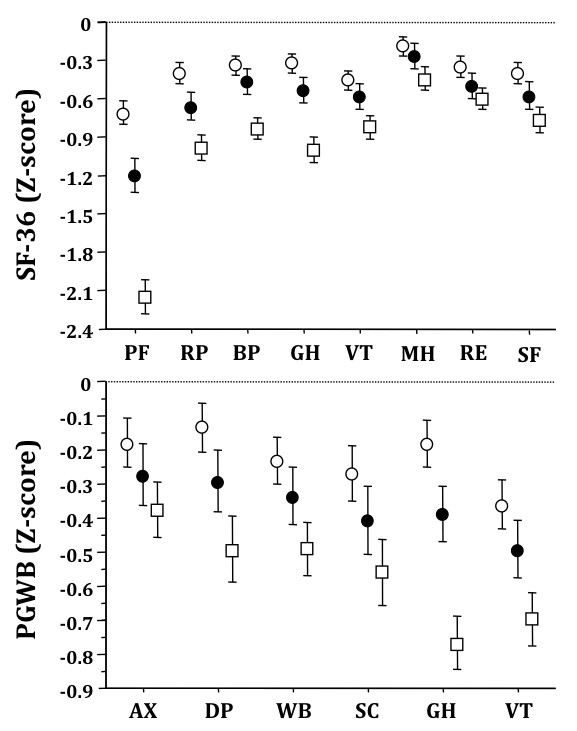
**Z-scores on Short Form-36 (upper panel) and Psychological General Well-being questionnaires in relation to obesity class (Class I (BMI, 30-34.9 kg/m^2^), open circles; Class II (BMI, 35-39.9), closed circles; Class III (BMI, ≥40), open squares).** Data are presented as means and 95% confidence intervals. Legend: for abbreviations, see Figure 1

Z-scores on SF-36 and PGWB in relation to obesity class are summarized in Figure 2. A systematic trend towards more severe impairment with increasing BMI (P < 0.001 was observed for all domains, except Anxiety at PGWB, P = 0.0024).

### Factors associated with poor HRQL

Logistic regression analysis was applied to identify factors associated with poor HRQL (Table 4). For both genders, the most significant factor was the presence of mental disease, as assessed by the composite score including both a reported previous history of psychological distress and a score at SCL-90 above the predefined cut-offs. This score was predictive of poor HROQL both in domains more closely associated with mental state and in those reflecting physical functioning. Data were confirmed by correlation analysis; the r coefficient of correlation between SCL-90 and individual Z-scores varied between -0.672 for Depressed mood in PGWB and -0.300 for Physical functioning in SF-36. Conversely, somatic disease, as expressed by the composite index, was associated with lower scores on the physical domains of SF-36, but had little impact on psychological domains, with the notable exception of social functioning. Among PGWB scales, only General Health appeared to be affected by somatic comorbidities in a relevant manner. No significant association of somatic index with ORWELL scores was observed, after adjustment for potential confounders.

**Table 4:** Association of clinical parameters with poor health-related quality of life.

	% +ve	BMI Class	Somatic disease	Mental disease	Binge eating	Weight history
**ORWELL-97**	24.8°	1.35 (1.03-1.75)^†^	1.17 (1.07-1.29)*	1.96 (1.74-2.21)*	1.64 (1.39-1.93)*	-----
**Short Form-36**						
Physical functioning	48.8	1.29 (1.00-1.66)^†^	1.22 (1.12-1.33)*	1.32 (1.18-1.47)*	-----	-----
Role-Physical	36.7	1.38 (1.09-1.75)^†^	1.25 (1.14-1.36)*	1.54 (1.38-1.72)*	1.28 (1.10-1.49)^†^	1.20 (1.04-1.38)^†^
Bodily pain	38.3°	-----	1.31 (1.20-1.43)*	1.47 (1.32-1.64)*	1.18 (1.01-1.36)^†^	-----
General health	36.2°	-----	1.36 (1.25-1.48)*	1.54 (1.37-1.72)*	1.29 (1.11-1.50)*	1.19 (1.04-1.37)^†^
Vitality	35.7°	-----	1.17 (1.08-1.28)*	1.89 (1.69-2.12)*	1.32 (1.14-1.54)*	-----
Role-Emotional	36.5°	-----	1.18 (1.08-1.28)*	1.89 (1.69-2.12)*	1.45 (1.25-1.69)*	-----
Mental health	25.4	1.34 (1.03-1.74)^†^	1.20 (1.09-1.32)*	1.98 (1.76-2.22)*	1.29 (1.10-1.52)*	-----
Social functioning	37.8°	1.32 (1.04-1.68)^†^	1.15 (1.05-1.25)^†^	2.03 (1.80-2.28)*	1.33 (1.14-1.54)*	1.16 (1.01-1.32)^†^
**Psychological General Well-Being**						
Depressed mood	21.9	-----	-----	2.12 (1.88-2.40)*	1.56 (1.31-1.84)*	-----
Anxiety	23.7	1.35 (1.02-1.76)^†^	1.17 (1.06-1.29)^†^	2.10 (1.86-2.36)*	1.32 (1.12-1.56)^†^	-----
Well-being	24.9	1.62 (1.25-2.11)*	1.13 (1.03-1.24)^†^	2.00 (1.78-2.25)*	1.35 (1.15-1.58)*	-----
Self-control	27.8°	-----	-----	2.05 (1.83-2.31)*	1.58 (1.35-1.85)*	-----
General health	28.4°	1.52 (1.18-1.94)*	1.30 (1.19-1.43)*	1.67 (1.49-1.87)*	1.46 (1.25-1.70)*	-----
Vitality	31.3	-----	1.21 (1.11-1.32)*	1.95 (1.73-2.18)*	1.43 (1.23-1.67)*	-----

A score of ORWELL-97 above the 75° percentile or a Z-score of individual domains of SF-36 and PGWB lower than -1.0 were the dependent variables. Data are presented as odds ratio (95% confidence intervals) for any 1-point increase in BMI class and in the scores of somatic and mental disease, binge eating and weight history (see Materials & Methods for calculations). All data are adjusted for age, gender and BMI.

°Significantly higher in females than in males (P < 0.05).

*P < 0.001; ^† ^P < 0.05 for the significance of association.

BMI class was systematically associated with poor HRQL in the ORWELL-97 score and in the physical domains of SF-36, namely in Physical functioning, but it had almost no effect on PGWB domains with the exception of General health. This association was confirmed at multivariate analysis, after adjustment for concurrent somatic and psychiatric diseases. In correlation analysis, the highest value was observed between BMI and the Z-score of Physical functioning (r = -0.405).

A BES score above the selected cut-offs was associated with poor HRQL in nearly all domains of HRQL measures, whereas a history of weight cycling was associated with poor HRQL only in a few domains of SF-36, namely in Role-Physical, General Health and Social Functioning.

In all models the Variance Inflation Factor was < 5, indicating the absence of multicolinearity.

## Discussion

In our study sample, obesity was associated with a relevant impairment of HRQL, in comparison with population norms, standardized for age and sex. This result is in keeping with previous reports of overweight-induced deterioration of HRQL across a wide age range [7,25-27]. The study sample was entirely composed of obese patients seeking medical treatment for weight loss and cannot be considered representative of the general population of obese subjects. In this respect, poor HRQL could be a motivation for referral and poorer scores are usually observed in clinic-based samples when compared with population-based surveys [27]. On the other hand, the study of these patients could provide a more accurate picture of obese individuals referring to specialized metabolic clinics, and provide relevant clues for treatment programs.

The study has several strengths. It was based on a very large sample of obese men and women in different centers, thus being representative of the "real world" of treatment-seeking obesity, outside specific research centers where a selection bias may be expected. As expected, obese women experienced a greater impairment of HRQL than their male counterparts. This confirms previous reports in clinic-based samples [6,15,25], among patients with chronic illness [5], and in population studies [28]. Gender differences in HRQL could be related to the higher prevalence of psychopathology among women [15,25,29], or to a greater cultural drive for thinness experienced by the female sex in Western societies [30].

Not surprisingly, subjects with higher BMI reported a greater impairment of HRQL, as previously reported [7,8]. This phenomenon can be partly due to the higher prevalence of concurrent somatic diseases and psychopathological disturbances in morbidly obese patients, when compared to individuals with lesser degrees of obesity. However, a greater impairment of HRQL in those with higher BMI persisted at multivariate analysis even after adjustment for somatic diseases, mental disorders, binge eating and weight cycling. A higher BMI appeared to affect mainly physical, rather than psychosocial, components of HRQL, suggesting that the functional impairment and physical discomfort determined by extreme overweight can have a major role in poor HRQL.

Somatic comorbidities, assessed through a score derived from Charlson's index [24], were associated with poorer scores on physical domains of HRQL instruments, but had little effect, after adjustment for confounders, on psychosocial domains. Concurrent somatic diseases also had a small impact on scores of the ORWELL-97 questionnaire, confirming its validity for obesity-related quality of life [15]. Conversely, psychopathological disturbances were associated with impairment of both physical and psychosocial domains of quality of life, even after adjustment for confounders. The presence of depressed mood and/or high levels of anxiety, which are the most common psychological disturbances observed in clinical samples of obese patients [31], can increase subjective distress induced by disease-related physical symptoms and functional impairment [15]. In the present sample, psychopathology was the most important predictor of quality of life among obese patients, in both psychosocial and physical domains. This result is partly in contrast with a previous survey in a small sample of obese patients undergoing bariatric surgery, where mental disorders appeared to affect psychosocial, but not physical domains of SF-36 [32]. Conflicting results can be attributed to differences in sample size (the previous sample being 18 times smaller than the one described in this study) or type of referral (surgery in the previous report *vs*. medical weight loss programs in the majority of centers of the present survey). In addition, the present study included obese subjects belonging to the whole spectrum of obesity classes, including a large group of subjects with obesity class III. These individuals are scarcely represented in medical settings, and may have a different psychopathological profile [33]. Finally, the definition of psychological disturbances in our study included not only a formal diagnosis of mental disorders, but also high scores on a questionnaire for general psychopathology, which could provide a more accurate description of the psychological status of patients at the time of HRQL assessment.

Binge eating disorder was previously reported to be associated with poor scores on disease-specific HRQL questionnaires [10,15]. This is consistent with the finding of a poorer perceived health status in patients with higher scores on the Binge Eating Scale. The association of binge eating with impaired HRQL can be partly mediated by higher BMI [34], a greater prevalence of mental disorders [31,34] and more frequent weight cycling in these cases. After adjustment for these potential confounders, binge eating was only marginally associated with some, but not all psychological domains of HRQL, without any impact on physical scales.

Finally, weight cycling is known to be associated with binge eating [34] and psychopathology [14], and with higher long-term morbidity and mortality [35-37], but its relationship with HRQL has never been demonstrated. In the present study, weight cycling was only associated with a few domains of quality of life, after adjustment for BMI class, somatic diseases, binge eating and psychopathology. It can be speculated that previous unsuccessful attempts at losing weight can negatively affect patients' confidence in the possibility to treat obesity effectively, thus making the psychological burden heavier and heavier. Accordingly, physicians should carefully test patients' motivation at entry into weight loss programs, considering that any treatment failure may be accompanied by a further deterioration of their HRQL. A definition of weight loss expectation and realistic treatment outcomes is pivotal to reduce the burden of disease associated with treatment failure [38].

The broad spectrum of questionnaires used in the study may also help identify which instruments should be preferred to detect impairment in HRQL in different settings. It is noteworthy that scores on both generic (SF-36, PGWB) and disease-specific (ORWELL-97) questionnaires appeared to be affected by the very same factors and in a similar manner. As expected, PGWB appeared to be more sensitive to psychological disturbances, while SF-36 and ORWELL-97 could detect to a greater extent the impact of physical conditions on HRQL. The choice of questionnaires in different settings should take into consideration the domains of greater interest (physical vs. psychological) in individual studies. The choice of instruments for the assessment of the effects of treatment on HRQL should also consider reliability, which is assumed to be greater for generic questionnaires, and sensitivity to change, which is thought to be superior for disease-specific questionnaires; these characteristics were not assessed in the present study.

## Conclusion

Our study has relevant clues to obesity treatment. HRQL is now considered a priority in the treatment of chronic diseases, and may be selected as clinical-relevant outcome in treatment programs [39]. The finding that psychopathological distress is the main determinant of poor HRQL makes psychiatric and psychological support essential in obesity centers. Only a multidisciplinary approach in weight management programs, addressing both mental and somatic disorders, is likely to reduce the burden of obesity in individual patients.

## Competing interests

The authors declare that they have no competing interests.

## Authors' contributions

EM drafted the manuscript and participated in study design; MLP drafted the manuscript and participated in study coordination; NV contributed to study discussion and performed the statistical analysis; CR conceived the study and participated in study design and coordination; GA conceived and designed the study; GM participated in study design and coordination, contributed to the statistical analysis, and wrote the manuscript; all the participants of the QUOVADIS Study Group collected the data. All authors read and approved the final manuscript.

## Note

A complete list of the participants in the QUOVADIS study has been previously published (Diab Nutr Metab 2003, **16**:115-124).

## References

[B1] Karlsson J Taft C Ryden A Sjostrom L Sullivan M Ten-year trends in health-related quality of life after surgical and conventional treatment for severe obesity: the SOS intervention study Int J Obes (Lond) 2007 31 8 1248 1261 10.1038/sj.ijo.0803573 17356530

[B2] Herpertz S Kielmann R Wolf AM Langkafel M Senf W Hebebrand J Does obesity surgery improve psychosocial functioning? A systematic review Int J Obes Relat Metab Disord 2003 27 11 1300 1314 10.1038/sj.ijo.0802410 14574339

[B3] Fontaine KR Barofsky I Obesity and health-related quality of life Obes Rev 2001 2 3 173 182 10.1046/j.1467-789x.2001.00032.x 12120102

[B4] Kushner RF Foster GD Obesity and quality of life Nutrition 2000 16 10 947 952 10.1016/S0899-9007(00)00404-4 11054600

[B5] Katz DA McHorney CA Atkinson RL Impact of obesity on health-related quality of life in patients with chronic illness J Gen Intern Med 2000 15 11 789 796 10.1046/j.1525-1497.2000.90906.x 11119171 PMC1495614

[B6] Kolotkin RL Crosby RD Kosloski KD Williams GR Development of a brief measure to assess quality of life in obesity Obes Res 2001 9 2 102 111 10.1038/oby.2001.13 11316344

[B7] Fontaine KR Cheskin LJ Barofsky I Health-related quality of life in obese persons seeking treatment J Fam Pract 1996 43 3 265 270 8797754

[B8] Kolotkin RL Crosby RD Williams GR Health-related quality of life varies among obese subgroups Obes Res 2002 10 8 748 756 10.1038/oby.2002.102 12181383

[B9] Marchesini G Bellini M Natale S Belsito C Isacco S Nuccitelli C Pasqui F Baraldi L Forlani G Melchionda N Psychiatric distress and health-related quality of life in obesity Diab Nutr Metab 2003 16 3 145 154 14635731

[B10] Rieger E Wilfley DE Stein RI Marino V Crow SJ A comparison of quality of life in obese individuals with and without binge eating disorder Int J Eat Disord 2005 37 3 234 240 10.1002/eat.20101 15822089

[B11] Marchesini G Natale S Chierici S Manini R Besteghi L Di Domizio S Sartini A Pasqui F Baraldi L Forlani G Effects of cognitive-behavioural therapy on health-related quality of life in obese subjects with and without binge eating disorder Int J Obes Relat Metab Disord 2002 26 9 1261 1267 10.1038/sj.ijo.0802073 12187405

[B12] Melchionda N Marchesini G Apolone G Cuzzolaro M Mannucci E Grossi E the QUOVADIS Study Group The QUOVADIS study. Features of obese Italian patients seeking treatment at specialist centers Diabetes Nutr Metab 2003 16 2 115 124 12846451

[B13] Maciejewski ML Patrick DL Williamson DF A structured review of randomized controlled trials of weight loss showed little improvement in health-related quality of life J Clin Epidemiol 2005 58 6 568 578 10.1016/j.jclinepi.2004.10.015 15878470

[B14] Marchesini G Cuzzolaro M Mannucci E Dalle Grave R Gennaro M Tomasi F Barantani EG Melchionda N Weight cycling in treatment-seeking obese persons: data from the QUOVADIS study Int J Obes Relat Metab Disord 2004 28 11 1456 1462 10.1038/sj.ijo.0802741 15314631

[B15] Mannucci E Ricca V Barciulli E Di Bernardo M Travaglini R Cabras PL Rotella CM Quality of life and overweight: the obesity related well-being (Orwell 97) questionnaire Addict Behav 1999 24 3 345 357 10.1016/S0306-4603(98)00055-0 10400274

[B16] McHorney CA Ware JE Jr Raczek AE The MOS 36-Item Short-Form Health Survey (SF-36): II. Psychometric and clinical tests of validity in measuring physical and mental health constructs Med Care 1993 31 3 247 263 10.1097/00005650-199303000-00006 8450681

[B17] Apolone G Mosconi P The Italian SF-36 Health Survey: translation, validation and norming J Clin Epidemiol 1998 51 11 1025 1036 10.1016/S0895-4356(98)00094-8 9817120

[B18] Dupuy HJ Wenger NK The psychological general well-being (PGWB) inventory Assessment of Quality of Life in Clinical Trials of Cardiovascular Therapies 1984 New York: Le Jacq Publications 170 183

[B19] Grossi E Mosconi P Groth N Niero M Apolone G Il Questionario Psychological General Well-Being. Versione Italiana 2002 Milano: Edizioni "Mario Negri"

[B20] Cohen J Statistical Power Analysis for the Behavioural Sciences 1977 8 New York: Academic Press

[B21] Kazis LE Anderson JJ Meenan RF Effect sizes for interpreting changes in health status Med Care 1989 27 3 Suppl S178 189 10.1097/00005650-198903001-00015 2646488

[B22] Gormally J Block S Daston S Rardin D The assessment of binge eating severity among obese persons Addict Behav 1982 7 1 47 55 10.1016/0306-4603(82)90024-7 7080884

[B23] Derogatis LR Cleary PA Confirmation of the dimensional structure of the SCL-90: a study in construct validity J Clin Psychol 1977 33 981 989 10.1002/1097-4679(197710)33:4<981::AID-JCLP2270330412>3.0.CO;2-0

[B24] Charlson ME Pompei P Ales KL MacKenzie CR A new method of classifying prognostic comorbidity in longitudinal studies: development and validation J Chronic Dis 1987 40 5 373 383 10.1016/0021-9681(87)90171-8 3558716

[B25] Sullivan M Karlsson J Sjöström L Backman L Bengtsson C Bouchard C Dahlgren S Jonsson E Larsson B Lindstedt S Swedish obese subjects (SOS) - an intervention study of obesity. Baseline evaluation and psychosocial functioning in the first 1743 subjects examined Int J Obesity Rel Metab Dis 1993 17 9 503 512 8220652

[B26] Borowiak E Kostka T Predictors of quality of life in older people living at home and in institutions Aging Clin Exp Res 2004 16 3 212 220 15462464 10.1007/BF03327386

[B27] Williams J Wake M Hesketh K Maher E Waters E Health-related quality of life of overweight and obese children JAMA 2005 293 1 70 76 10.1001/jama.293.1.70 15632338

[B28] Burns CM Tijhuis MA Seidell JC The relationship between quality of life and perceived body weight and dieting history in Dutch men and women Int J Obes Relat Metab Disord 2001 25 9 1386 1392 10.1038/sj.ijo.0801714 11571604

[B29] Weissman MM Klerman GL Sex differences and the epidemiology of depression Arch Gen Psychiatry 1977 34 1 98 111 319772 10.1001/archpsyc.1977.01770130100011

[B30] Foster GD Wadden TA Blackburn GL, Kanders BD The psychology of obesity, weight loss, and weight regain: research and clinical findings Obesity: Pathophysiology, Psychology and Treatment 1994 New York: Chapman & Hall 140 159

[B31] Ricca V Mannucci E Moretti S Di Bernardo M Zucchi T Cabras PL Rotella CM Screening for binge eating disorder in obese outpatients Compr Psychiatry 2000 41 2 111 115 10.1016/S0010-440X(00)90143-3 10741889

[B32] Callegari A Michelini I Sguazzin C Catona A Klersy C Efficacy of the SF-36 questionnaire in identifying obese patients with psychological discomfort Obes Surg 2005 15 2 254 260 10.1381/0960892053268255 15802070

[B33] Petroni ML Villanova N Avagnina S Fusco MA Fatati G Compare A Marchesini G Psychological distress in morbid obesity in relation to weight history Obes Surg 2007 17 3 391 399 10.1007/s11695-007-9069-3 17546849

[B34] Marcus MD Brownell KD, Fairburn CG Binge eating and obesity Eating Disorders and Obesity 1995 New York: Guildford 441 445

[B35] Blair SN Shaten J Brownell K Collins G Lissner L Body weight change, all-cause mortality, and cause-specific mortality in the Multiple Risk Factor Intervention Trial Ann Intern Med 1993 119 7 Pt 2 749 757 8363210 10.7326/0003-4819-119-7_part_2-199310011-00024

[B36] Lee IM Paffenbarger RS Jr Change in body weight and longevity JAMA 1992 268 15 2045 2049 10.1001/jama.268.15.2045 1404740

[B37] Lissner L Odell PM D'Agostino RB Stokes J Kreger BE Belanger AJ Brownell KD Variability of body weight and health outcomes in the Framingham population N Engl J Med 1991 324 26 1839 1844 10.1056/NEJM199106273242602 2041550

[B38] Dalle Grave R Calugi S Molinari E Petroni ML Bondi M Compare A Marchesini G Weight loss expectations in obese patients and treatment attrition: an observational multicenter study Obes Res 2005 13 11 1961 1969 10.1038/oby.2005.241 16339128

[B39] Apolone G De Carli G Brunetti M Garattini S Health-related quality of life (HR-QOL) and regulatory issues. An assessment of the European Agency for the Evaluation of Medicinal Products (EMEA) recommendations on the use of HR-QOL measures in drug approval Pharmacoeconomics 2001 19 2 187 195 10.2165/00019053-200119020-00005 11284382

